# Supplementary material for: Association between Skeletal Muscle Loss and the Response to Neoadjuvant Chemotherapy for Breast Cancer
Source: Cancers (Basel). 2021 Apr 9;13(8):1806. doi: 10.3390/cancers13081806 (PMC8070318; doi:10.3390/cancers13081806)
Supplement: Supplementary file 1 [file cancers-13-01806-s001.zip › cancers-1137471-supplementary.pdf]

Supplementary Materials: Association Between Skeletal Muscle Loss and the Response to Neoadjuvant Chemotherapy for Breast Cancer

Byung Min Lee, Yeona Cho, Jun Won Kim, Sung Gwe Ahn, Jee Hung Kim, Hei Cheul Jeung, Joon Jeong and Ik Jae Lee

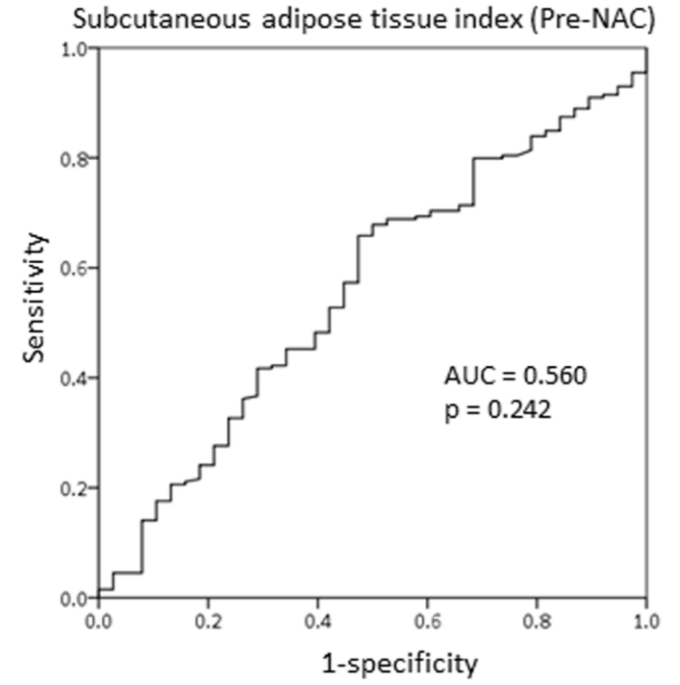

Figure S1. Receiver operating characteristic curve of the subcutaneous adipose tissue index at the T4 spine prior to the initiation of neoadjuvant chemotherapy.

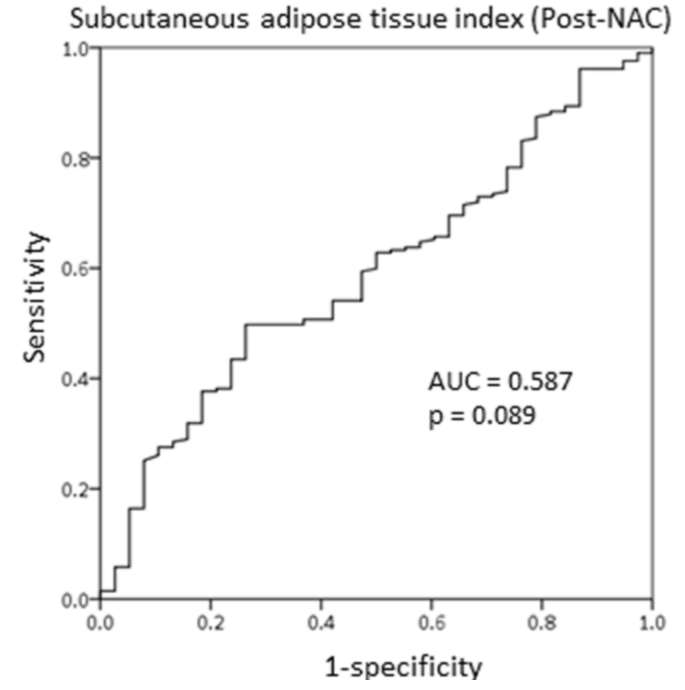

Figure S2. Receiver operating characteristic curve of T4 spine subcutaneous adipose tissue index after completion of neoadjuvant chemotherapy.
